# M^6^A Regulates Intramuscular Fat Deposition in Rabbits Through *LPL*/3-Methyl-L-Histidine/Pathways

**DOI:** 10.3390/ani16111646

**Published:** 2026-05-28

**Authors:** Gang Luo, Jihao Le, Xiaoming Mao, Tongtong Xue, Zhanjun Ren

**Affiliations:** 1 College of Animal Science, Fujian Agriculture and Forestry University, Fuzhou 350000, China; 2 College of Animal Science and Technology, Northwest A&F University, Yangling 712100, China

**Keywords:** *METTL3*, M^6^A, *LPL*, Rex rabbits, IMF

## Abstract

The flavor of rabbit meat has always been a major factor hindering the development of the rabbit industry. One of the main factors affecting the flavor of rabbit meat is intramuscular fat. N6-methyladenosine (m^6^A) regulates multiple aspects of the physiology of animals. In this study, qRT-PCR and m^6^A-qPCR were used to identify genes and methylation levels. AAV virus was used as a vector to overexpress genes. To explore the regulatory mechanism of m^6^A on intramuscular fat in rabbits, we first explored the regulation of the *LPL* gene of rabbits by m^6^A at the cellular level using interfering RNA. Subsequently, we further validated the mechanism and explored the regulation of metabolites by *LPL* genes in living dorsal muscles. The results demonstrate that *METTL3* inhibited *LPL* expression through m^6^A modification under the recognition of YTHDF2 in adipocytes and muscles. *LPL* promotes adipocyte differentiation and intramuscular fat deposition. In addition, *LPL* regulates intramuscular fat deposition through L-Glutamine/multiple pathways and 3-Methyl-L-histidine. This study confirms that m^6^A can affect the expression of the *LPL* gene in rabbits, thereby regulating the IMF of rabbit meat by L-Glutamine/multiple pathways and 3-Methyl-L-histidine. This study lays the molecular foundation for cultivating high-quality rabbit meat.

## Simple Summary

Rabbit meat is known as a health and beauty meat due to its high protein, high lysine, and high phospholipid content, among other characteristics. But the growth rate of rabbit meat consumption has been relatively slow in recent years. Low intramuscular fat and low flavor compounds are one of the main reasons for the low consumption of rabbit meat. M^6^A is a common RNA modification involved in the regulation of multiple aspects such as gene transcription, translation, and degradation. In this study, we first explored the regulation of m^6^A modification on *LPL* gene expression and methylation levels at the cellular level. Subsequently, the regulatory mechanisms in cells were validated in vivo. Meanwhile, we also explored the effects of overexpression of the LPL gene on fatty acids and amino acids in rabbits. Through summary and analysis, we found that m^6^A can affect the composition of fatty acids and amino acids in rabbit muscles by modifying the *LPL* gene. This study laid a molecular foundation for the improvement of rabbit meat quality by altering the m^6^A modification of genes.

## 1. Introduction

Rabbits are a special economic animal that can convert agricultural and sideline products and weeds into high-quality meat products. Rabbit farming not only contributes to national food security but also effectively reduces environmental pollution. However, the low intramuscular fat content of rabbits reduces the consumption of rabbit meat and slows down the development of the rabbit industry. Fat deposition is characterized as increased cell size (hypertrophy) and increased cell numbers at the cellular level. Adipose tissue deposition is closely related to the meat quality and meat flavor of domestic animals. Drip loss, cooked meat rate, intramuscular fat content (IMF) and the flavor of meat are the main indicators for evaluating meat quality [1]. Amino acids are also important precursors of freshness. Glycine, aspartic acid, arginine, glutamic acid, and alanine are amino acids that belong to the category of freshness, whose composition and content directly affect the quality and freshness of meat [2]. In addition, n-3 polyunsaturated fatty acids play important roles in the taste of meat [2,3]. Improvement of the IMF not only increases sensory and health properties but also improves overall meat quality [4]. A low IMF content and insufficient flavor of rabbit meat have seriously affected the development of the rabbit industry. Therefore, exploring the molecular regulatory mechanisms of rabbit meat quality and flavor is of great economic significance for the development of the rabbit industry.

N6-methylation on adenosine (m^6^A) is the most prevalent internal mRNA modification in eukaryotes [5,6]. RNA m^6^A modification can be catalyzed by m^6^A writer proteins (*METTL3*/*METTL14*/*WTAP*) [7,8], recognized by m^6^A reader proteins (*YTHDF1-3*), and removed by m^6^A eraser proteins (*FTO* and *ALKBH5*) [9]. Recently, it has been proposed that m^6^A regulates adipogenesis through mediating mRNA splicing [10]. Methyltransferase-like 3 (*METTL3*), a key RNA methyltransferase, has been demonstrated to regulate neurogenesis [11], spermatogenesis [12,13], early embryonic development [14] and stem cell pluripotency in mice [14,15]. In addition, a study shows that *METTL3* regulates the differentiation of preadipocytes through m^6^A modification under the recognition of YTHDF2 [16]. In our previous study, we found multiple m^6^A modification sites on the *LPL* gene of rabbits [17].

Lipoprotein lipase (*LPL*) mainly produces free fatty acids, chylomicrons residues and intermediate density lipoproteins in circulating blood [18,19]. *LPL* can regulate the selective absorption of lipoproteins related to lipids without the absorption of lipoproteins [20]. According to the current research, the *LPL* gene can regulate the development of adipose tissue [21]. The study showed that the expression of the *LPL* gene is consistent with the trend of changes in fat deposition in subcutaneous adipose tissue [22]. In addition, *LPL* not only regulates the differentiation and maturation of adipocytes but also influences the distribution of TG in fat and muscle [23]. These results indicate that *LPL* may regulate the meat flavor of animals. However, whether *METTL3*-mediated m^6^A modification of mRNA regulates *LPL* expression has not been investigated. At the same time, *LPL* can also affect the generation of amino acids and fatty acids in animals’ bodies [24,25]. One study has found that replacing three polar amino acid residues (hstidine46 L-Glutamine50 and L-Glutamine53) in mouse sequences with alanine can eliminate the inhibitory effect of *ANGPTL4* on *LPL* [26]. However, the regulatory role of LPL on A and B is still unclear.

In this study, we explored the regulatory mode of *METTL3* on *LPL* genes and the regulation of *LPL* on preadipocyte differentiation. Then, we validated the effect of *METTL3* on *LPL* through m^6^A modification under the recognition of *YTHDF2* in the longissimus lumborum muscle of rabbits. Finally, we explored the regulation of the *LPL* gene on intramuscular fat, amino acids, and fatty acid content. Our study showed that m^6^A regulates intramuscular fat deposition in rabbits through *LPL*/3-Methyl-L-histidine/pathways.

## 2. Materials and Methods

### 2.1. Ethical Statement

This study was conducted and approved by the Institutional Animal Care and Use Committee of the College of Animal Science and Technology, FAFU, Fuzhou (Permit Number: PZCASFAFU22003).

### 2.2. Animals and Tissue Collection

Preadipocyte was isolated and cultured from perirenal adipose tissues of 0-day-old Rex rabbits. Longissimus lumborum and blood were collected from six 35-day-old, six 75-day-old and six 165-day-old Rex rabbits. Thirty 123-day-old Rex rabbits were used for overexpression experiments in vivo. All rabbits used in this study were male. (Male rabbits improve a population much faster than females, so male rabbits were used.) All rabbits were taken from the Xinjin Otter Rabbit Farm in Sichuan, and then we obtained 0-day-old rabbits through breeding. All rabbits were housed in single cages and fed regularly under natural ventilation. The temperature during the experiment was between 20 and 30 °C. During the entire experimental period, all rabbits drank standard drinking water and consumed regular commodity diets. Ear vein blood was collected before slaughtering rabbits. All animals were slaughtered in the laboratory of Fujian Agriculture and Forestry University with minimal harm.

### 2.3. Cell Isolation, Culture, and Induction of Adipogenesis

Perirenal fat was isolated from newborn rabbits and washed in PBS. After the tissue was cut into pieces, we used type 1 collagenase to digest in a 37 °C water bath for about 1 h. We used a growth medium to wash the centrifuge tube and turned liquid into a culture bottle to cultivate cells. The culture bottle was placed in a 37 °C sterile incubator for cultivation, and the solution was changed after 48 h. When the cell growth density reached 80%, we used an induction distribution solution to induce adipocyte differentiation. Cell experiments used repeated techniques.

### 2.4. Cell Transfection

Lipofectamine 2000 (Invitrogen, Carlsbad, CA, USA) was used to transfect preadipocyte cells. The cells were harvested at different time intervals after transfection and used to study adipogenic differentiation.

### 2.5. RNA Extraction, cDNA Synthesis and qRT-PCR

Total RNA was extracted using TRIzol reagent. We used the NanoDrop 2000 spectrophotometer (Thermo, Waltham, MA, USA) to detect RNA. cDNA was synthesized by the PrimeScript RT Reagent Kit (Takara, Kusatsu, Japan), and SYBR Premix Ex Taq II (TliRNase H Plus) (Catalog No. RR820A; Takara) was performed qPCR. Primers are in Table 1.

### 2.6. m^6^A-qPCR

We used the Magna MeRIP m^6^A Kit (Millipore, Billerica, MA, USA) to examine m^6^A modifications on individual genes. Briefly, total RNA was fragmented by metal–ion-induced fragmentation. Then, RNA fragments were incubated with m^6^A antibody (#MABE1006, included in the kit)-conjugated Protein A/G magnetic beads. Methylated RNAs were eluted and were analyzed by qPCR along with the MeRIPed RNAs.

### 2.7. Oil Red O Staining and Determination of Triglyceride Content

Adipocytes were washed with phosphate-buffered saline (PBS) and fixed in 4% paraformaldehyde for 30 min. Cells were stained with Oil Red O and observed under a phase contrast microscope. Intracellular triglyceride (TG) content was quantified with the TG Assay Kit (Applygen, Beijing, China).

### 2.8. Measurement of LPL in Blood

Concentrations of LPL were determined using LPL ELISA kits (USCN Life Science Inc., Wuhan, China). The actual sensitivities of LPL are typically <0.1 ng/mL. The mean intra- and inter-assay coefficient of variation for each LPL was <10 and <15%, respectively. Serial dilutions of plasma samples showed good linearity (differed by 20% or less) in the calculated concentrations of LPL. Each LPL had no significant cross-reactivity or interference with respective analogs.

### 2.9. Measurement of IMF

IMF was measured using ether. Briefly, the meat sample was chopped, dehydrated, and crushed. About 1.0 g of treated sample (mf) was accurately weighed into a filter paper cylinder and then dehydrated to a constant amount in an oven at 105 °C so that the total mass was mf1. Subsequently, the sample was treated in a Soxhlet extractor and was dried in an oven at 105 °C to a constant amount (mf2). The formula of intramuscular fat content (w) is as follows: w = (mf1 − mf2)/mf × 100%.

### 2.10. Recombinant AAV Injection

AAV9 was selected as the vector for this study. Recombinant AAV formation requires vector construction, packaging, collection, purification, titer determination, and cell infection testing before it can be used in animal experiments (Hanheng Biotechnology corporation, Shanghai, China). The carrier was digested by a restriction enzyme and then recovered and purified by agarose gel. PCR obtains suitable target fragments, which are then ligated to a vector and cultured. Finally, monoclonal traveling colonies were used for validation and sequencing to establish overexpression vector plasmids. In this study, we synthesized recombinant AAVs to overexpress *METTL3*, *YTHDF2*, and *LPL* genes using AAV9. Each rabbit was selected to inject recombinant AAV at 5 fixed points, and 10 μL (1 × 10^12^) per point was injected. The virus was injected into the rabbit’s longest dorsal muscle at a distance of 1 cm from the spine and 2 cm from the buttocks. Each session lasted for 21 days, with 2 samples taken after each session. We grouped 6 rabbits and injected each rabbit twice (123-day-olds and 144-day-olds).

### 2.11. Determination of Amino Acid and Fatty Acid Content

We measured the content of amino acids and fatty acids by targeting metabolomics. The sample was added to a centrifuge tube containing steel balls and 1 mL of a solution (methanol, acetonitrile, and water) for 30 s. Then, we repeated the steps of 45 Hz homogenization for 4 min and ultrasound for 5 min under ice-water-bath conditions three times. The supernatant was obtained after being left to stand, centrifuged, and filtered. UHPLC-MS/MS was used to analyze the supernatant of metabolites. The SCIEX Analyst Work Station Software (Version 1.7.2) and Sciex OS 2.0.1 were employed for MRM data acquisition and processing. Metabolomics measurement and analysis were conducted at Baimaike Biotechnology Co., Ltd. (Beijing, China). It is generally believed that metabolites with VIP ≥ 1 have significant differences. The confidence interval is 95%. When Q2Y > 0.5, it can be considered an effective model, and when Q2Y > 0.9, it is an excellent model. The difference in metabolites between the control group and the experimental group was more than 1-fold, with a fold change difference ≥ 1 considered significant. *p* ≤ 0.05 is considered significant.

### 2.12. Statistical Analysis

The data was analyzed by GraphPad Prism7 (GraphPad Software, La Jolla, CA, USA). We used two-tail Student’s *t*-test and one-way analysis of variance in this study. *p* < 0.05 and *p* < 0.01 were deemed to be significant and highly significant, respectively.

## 3. Results

### 3.1. The Expression of METTL3 and LPL at Different Stages and the Methylation Modification of the LPL Gene in Different Tissues

**Figure 1:**
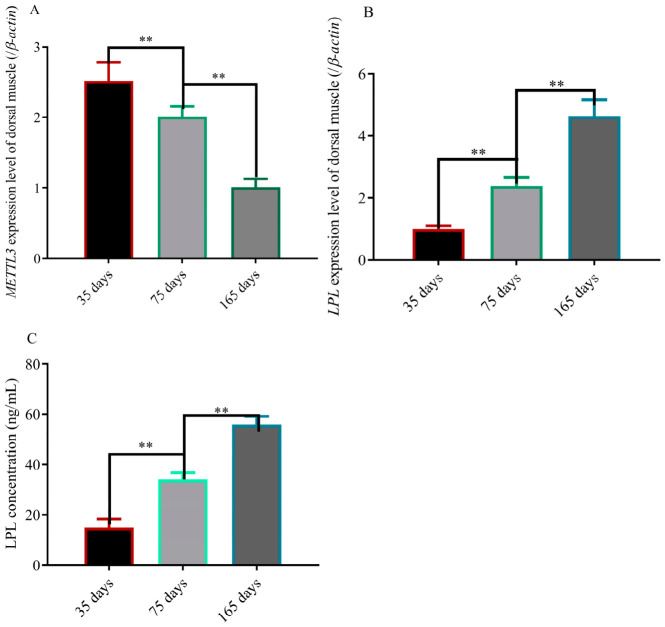
Expression of *LPL* and *METTL3* genes in Rex rabbits at different stages. (**A**) The expression of *METTL3* in muscle tissues; (**B**) the expression of *LPL* in muscle tissues; (**C**) content of *LPL* in blood. (“**”, *p* < 0.01).

**Figure 2:**
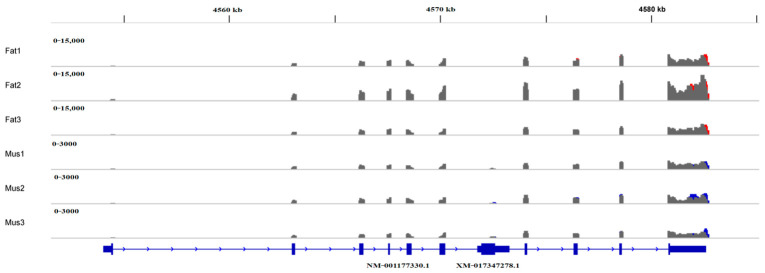
Modification sites of m^6^A on the *LPL* gene in fat and muscle tissues [17].

To explore the mechanism of intramuscular fat deposition, we detected the expression of *METTL3* and *LPL* at different ages. We found the expression level of *METTL3* in muscles at 35 days of age to be significantly higher than that of muscles at 75 days of age, and the expression level of *METTL3* in muscles at 75 days of age is significantly higher than that of muscles at 165 days of age (*p* < 0.01) (Figure 1A). On the contrary, the expression level of *LPL* in muscles and the content of LPL in the blood at 35 days of age is significantly lower than those at 75 days of age, and the expression level of *LPL* in muscles and the content of LPL in the blood at 75 days of age is significantly lower than those at 165 days of age (*p* < 0.01) (Figure 1B,C). As shown in Figure 2, we found multiple methylation modification sites on the mRNA of the *LPL* gene, and there are differences between muscle and fat tissues.

### 3.2. METTL3 and YTHDF2 Synergistically Regulate the Expression and Methylation Level of LPL

**Figure 3:**
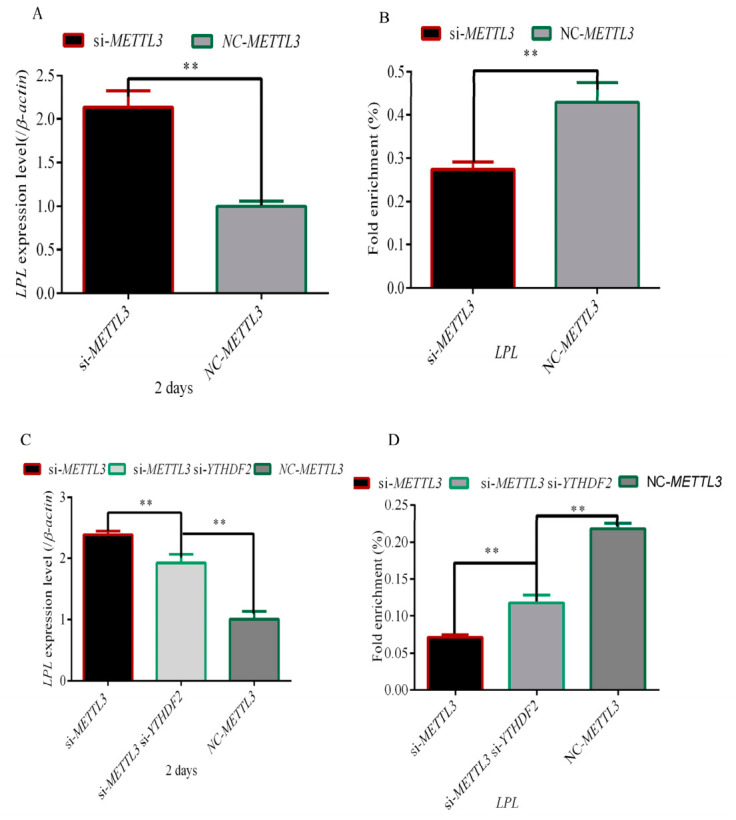
*METTL3* downregulates the expression of *LPL* through *YTHDF2* during the differentiation of preadipocytes. (**A**) The mRNA expression of *LPL* after interfering with *METTL3*; (**B**) methylation level of *LPL* gene after interfering with *METTL3*; (**C**) the mRNA expression of *LPL* after interfering with *METTL3* and *YTHDF2*; (**D**) methylation level of *LPL* gene after interfering with *METTL3* and *YTHDF2* (“**”, *p* < 0.01).

In order to explore the regulation mechanism of the *METTL3* gene on *LPL*, we detected the expression level of *LPL* after interfering with the *METTL3* gene. *LPL* expression significantly increased after interference with *METTL3* (*p* < 0.01) (Figure 3A). However, the methylation level of the *LPL* gene decreased after interference with *METTL3* (*p* < 0.01) (Figure 3B). Our results demonstrate that loss of *YTHDF2* decreased mRNA levels of *LPL* in *METTL3*-depleted cells (*p* < 0.01) (Figure 3C). In addition, interference of YTHDF2 could partially increase methylation of *LPL* caused by *METTL3* interference (*p* < 0.01) (Figure 3D).

### 3.3. Downregulation of LPL Expression Inhibited Rabbit Preadipocyte Differentiation

**Figure 4:**
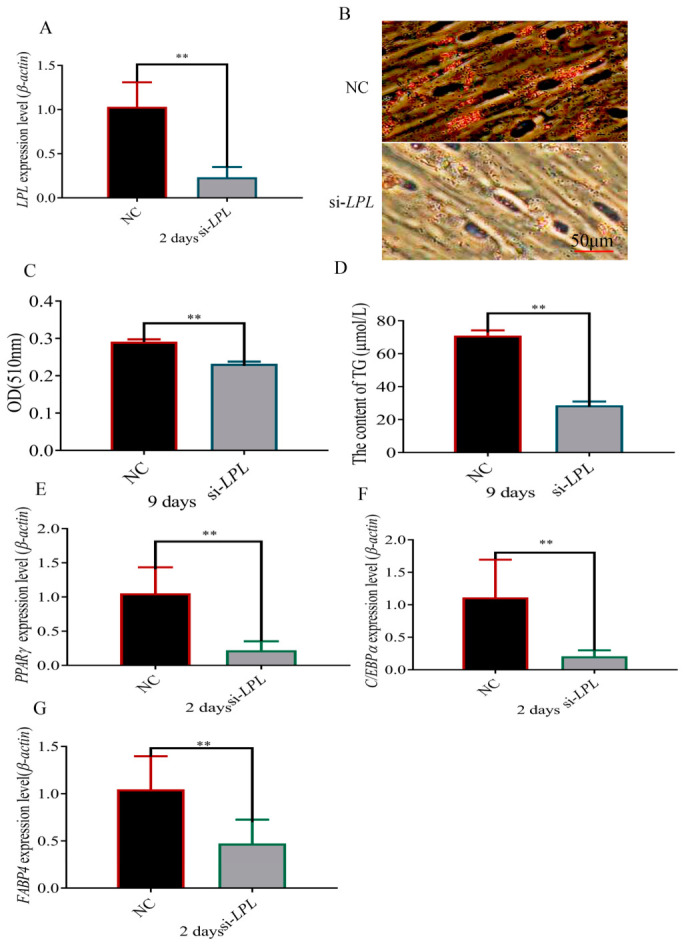
*LPL* upregulates differentiation of preadipocytes. (**A**) The mRNA expression of *LPL* after interfering with *LPL*; (**B**) oil red staining after interfering with *LPL*; (**C**,**D**) fat droplets and triglyceride content after interfering with *LPL*; (**E**–**G**) the mRNA expression of *PPARγ*, *C/EBPα* and *FABP4* after interfering with *LPL* (“**”, *p* < 0.01).

To further confirm whether *LPL* is involved in the differentiation of rabbit preadipocytes, si-LPL was utilized to cause interference with endogenous *LPL*. After interfering with the *LPL* gene, we found that the mRNA and protein levels of the *LPL* gene were significantly decreased after adipogenic induction differentiation for 2 days (*p* < 0.01) (Figure 4A). Then, we detected fat droplet accumulation by the oil red O staining assay after adipogenic induction differentiation for 9 days, and the results show that the fat droplet accumulation was reduced in the si-LPL group (*p* < 0.01) (Figure 4B,C). In addition, TG significantly decreased after interfering with *LPL* (*p* < 0.01) (Figure 4D). When *LPL* was interfered with, the mRNA levels of *PPARγ*, *C/EBPα* and *FABP4* rapidly decreased on the second day after transfection (*p* < 0.01) (Figure 4E–G).

### 3.4. METTL3 Mediates Lipogenesis in an m^6^A-YTHDF2-Dependent Manner in Rex Rabbits In Vivo

**Figure 5:**
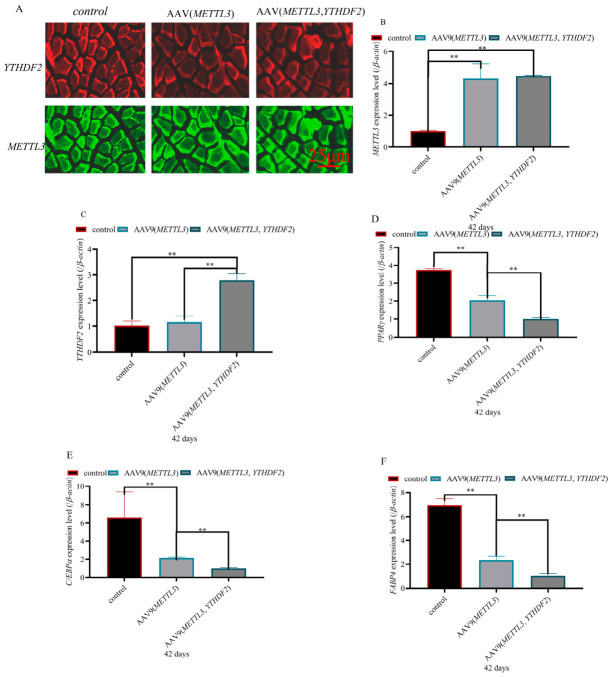
*METTL3* downregulates adipogenesis through *YTHDF2*. (**A**) Slicing of muscle tissues after overexpression of *METTL3* and *YTHDF2*; (**B**) the mRNA expression of *METTL3* after overexpression of *METTL3* and *YTHDF2*; (**C**) the mRNA expression of *YTHDF2* after overexpression of *METTL3* and *YTHDF2*; (**D**–**F**) the mRNA expression of *PPARγ*, *C/EBPα* and *FABP4* after overexpression of *METTL3* and *YTHDF2* (“**”, *p* < 0.01).

To ascertain whether *YTHDF2* is a major contributor to the function of *METTL3* in adipogenesis, we overexpressed *METTL3* and *YTHDF2* in vivo. As shown in Figure 5A, the fluorescence carried by both the *METTL3* and *YTHDF2* genes is fully stimulated in muscle tissues. The mRNA expression levels of *METTL3* and *YTHDF2* indicated that *METTL3* and *YTHDF2* were successfully overexpressed in muscles (Figure 5B,C). However, the expression levels of *PPARγ*, *C/EBPα* and *FABP4* were significantly reduced after overexpression of *METTL3* (*p* < 0.01) (Figure 5D–F). In addition, *FABP4*, *C/EBPα* and *PPARγ* expressions after overexpressing *METTL3* and *YTHDF2* simultaneously were lower than those of overexpressing *METTL3* (*p* < 0.01) (Figure 5D–F).

### 3.5. METTL3 Downregulated the Expression of LPL in an m^6^A-YTHDF2-Dependent Manner in Rex Rabbits In Vivo

**Figure 6:**
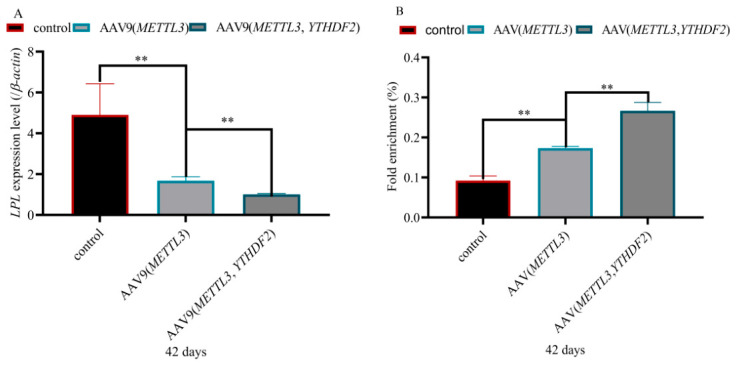
*METTL3* downregulates *LPL* expression through *YTHDF2*. (**A**) The mRNA expression of *LPL* after overexpression of *METTL3* and *YTHDF2*; (**B**) methylation level of *LPL* after overexpression of *METTL3* and *YTHDF2* (“**”, *p* < 0.01).

In the living dorsal muscles of Rex rabbits, the mRNA expression of the *LPL* gene is downregulated after overexpression of *METTL3* and is lowest when both *METTL3* and *YTHDF2* are overexpressed simultaneously (Figure 6A). On the contrary, the methylation level of the *LPL* gene increased after overexpression of *METTL3*, and the level further increased after simultaneous overexpression of *METTL3* and *YTHDF2* (Figure 6B).

### 3.6. LPL Upregulated Lipogenesis in Rex Rabbits In Vivo

**Figure 7:**
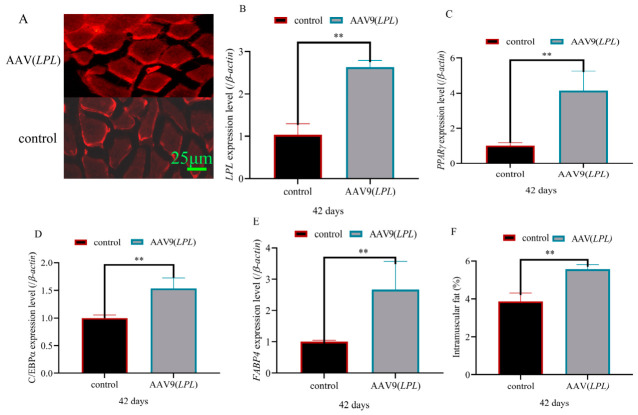
*LPL* upregulates adipogenesis. (**A**) Slicing of muscle tissues after overexpression of *LPL*; (**B**) the mRNA expression of *LPL* after overexpression of *LPL*; (**C**–**E**) the mRNA expression of *PPARγ*, *C/EBPα* and *FABP4* after overexpression of *LPL*; (**F**) IMF content after overexpression of *LPL* (“**”, *p* ≤ 0.01).

To determine the regulation of the *LPL* gene on adipogenesis in vivo, we overexpressed the *LPL* gene in the longissimus lumborum muscle of Rex rabbits. The excitation of red fluorescence indicated that *LPL* had been expressed in the dorsal muscles of Rex rabbits (Figure 7A). The mRNA level of the *LPL* gene was significantly increased after overexpression of *LPL* gene (*p* < 0.01) (Figure 7B). *FABP4*, *C/EBPα* and *PPARγ* expression were significantly increased after overexpression of *LPL* (*p* < 0.01) (Figure 7C–E).

### 3.7. LPL Regulated Composition of Amino Acid and Fatty Acid Lipogenesis in Rex Rabbits In Vivo

**Figure 8:**
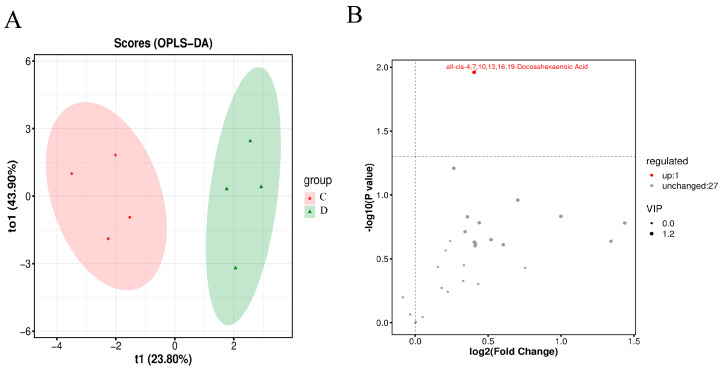
Overview of differential fatty acids. (**A**) Results of orthogonal partial least squares discriminant analysis; (**B**) volcanic diagram of differential fatty acids.

**Figure 9:**
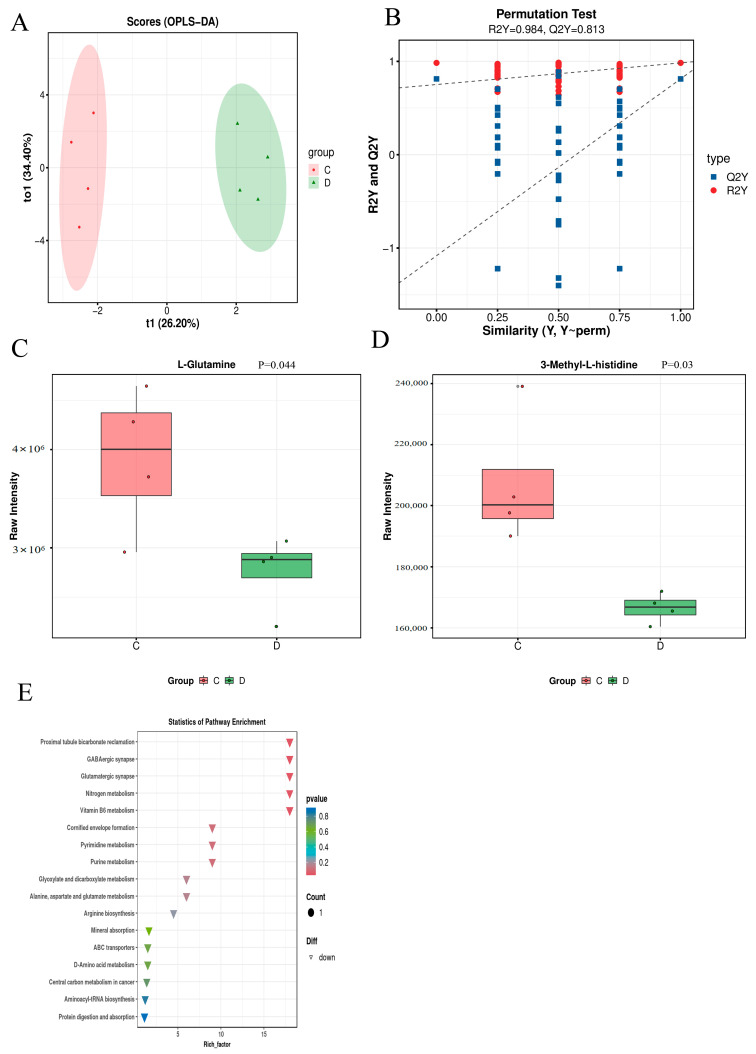
Overview of differential amino acids. (**A**) Results of orthogonal partial least squares discriminant analysis; (**B**) results of orthogonal partial least squares discriminant analysis (permutation); (**C**) box line diagram of L-Glutamine; (**D**) box line diagram of 3-Methyl-L-histidine; (**E**) signal pathway enrichment map.

To explore the regulation of *LPL* on fatty acids in rabbits, we measured the content of fatty acids in muscle tissues after overexpression of the *LPL* gene in rabbits. As shown in Figure 8A, we found a significant difference between control group and experimental group samples. The results of fatty acid content indicate that all-dis-4,7,10,13,16.19-Deoosahebonoie Acid is upregulated after overexpression of the *LPL* gene (Figure 8B). At the same time, we found that the analysis results of intra-group repeatability and inter-group differences in amino acid determination samples were also good (Figure 9A,B). In addition, we found that the content of L-Glutamine and 3-Methyl-L-histidine was significantly downregulated in the experimental group (*p* < 0.05) (Figure 9C,D). Through enrichment of signaling pathways, it was found that L-Glutamine is involved in the regulation of 17 signaling pathways, among which Vitamin B6 metabolism and D-Amino acid metabolism, Arginine biosynthesis, alanine, aspartate and glutamate metabolism, and Glutamatergic synapse play important roles in the process of fat deposition.

### 3.8. LPL Regulated IMF Through Metabolites

**Figure 10:**
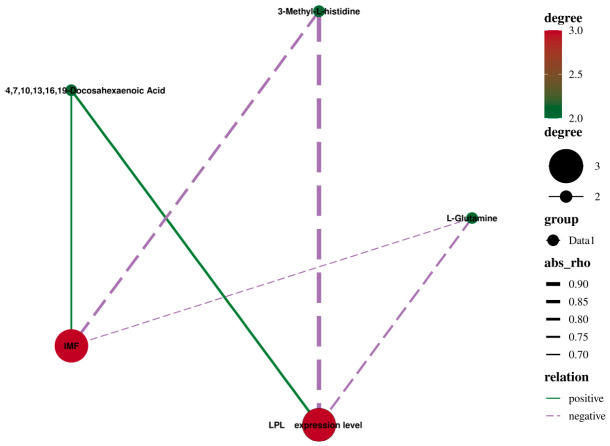
Correlation network diagram of *LPL* expression–fatty acids and amino acids–IMF.

As shown in Figure 10, correlation analysis results show that the expression level of *LPL* was significantly positively correlated with all-dis-4,7,10,13,16.19-Deoosahebonoie Acid but significantly negatively correlated with L-Glutamine (*p* < 0.05). In addition, the expression of *LPL* was significantly negatively correlated with the content of 3-Methyl-L-histidine (*p* < 0.01). Meanwhile, we also found a significant negative correlation between the content of 3-Methyl-L-histidine and the content of IMF (*p* < 0.05). So, we speculate that *LPL* may regulate the content of IMF through 3-Methyl-L-histidine.

## 4. Discussion

*METTL3*, as a methyltransferase, catalyzes the formation of m^6^A and plays important roles in various biological processes [7,13]. Studies reported that *METTL3* interacts with translation initiation machinery to promote translation of a subset of m^6^A containing mRNAs independently of its methyltransferase activity and m^6^A readers [27]. However, whether *METTL3* plays roles in adipogenesis and the underlying mechanisms are still largely unknown. In this study, *METTL3* expression decreased during the growth process of rabbits. Studies showed that the content of IMF increased during the growth process of rabbits [28]. In summary, *METTL3* may regulate the deposition of IMF through methylation modification. In our previous study, we found that the *LPL* gene was modified by m^6^A in both rabbit muscle and adipose tissue [17]. At the same time, we found that the expression of the *LPL* gene in muscles and the content of LPL in blood increased with an increase in rabbit age.

In order to explore the regulation of *LPL* by *METTL3*, we found that interference with *METTL3* during preadipocyte differentiation resulted in a decrease in *LPL* gene expression but an increase in methylation levels. These results indicate that *METTL3* can regulate the expression of *LPL* genes through m^6^A modification. YTHDF2 is a methylation recognition enzyme, and *METTL3* can regulate the expression of the *PCK2* gene through YTHDF2’s recognition [16]. Similarly, we also found that YTHDF2 can alter the regulation of *METTL3* on *LPL* gene methylation and expression levels by identifying the m^6^A site in this study.

To explore the relationship between the *LPL* gene and adipocyte differentiation, we interfered with the expression of the *LPL* gene. The triglyceride content was found to be less, and lipid droplets were smaller after transfection with si-LPL. The downregulation of *LPL* decreased the mRNA expression of *FABP4*, *PPARγ*, and *CEBPα*. At the same time, the protein level of *PPARγ* also decreased significantly. The role of the lipoprotein lipase (*LPL*) gene in adipogenesis has been previously reported, and expression of *LPL* messenger RNA reached a stable level in mature adipocytes in the early stages of adipogenesis, which indicates that *LPL* is one of the critical factors in the process of adipogenic differentiation [29]. Studies have also shown that *LPL* plays an important role in lipid metabolism, obesity, insulin effect and weight regulation, whose mRNA expression level, protein concentration and catalytic activity can be used as markers of adipocyte differentiation [29]. These results indicate that the *LPL* gene can promote adipocyte differentiation.

Adipocyte differentiation and proliferation are the main causes of fat deposition. In order to explore the regulatory mechanism of the *LPL* gene on fat in muscles, we overexpressed *METTL3* and *LPL* genes in living rabbits and found that they can both promote fat deposition. In addition, *METTL3* can also regulate *LPL* gene expression through *YTHDF2* in living rabbits. These results indicate that *METTL3*, *LPL*, and *YTHDF2* have the same regulatory mechanism for fat deposition in preadipocytes and intramuscular fat.

*LPL* participates in the PPAR signaling pathway and is associated with lipid transfer and metabolism [30]. In addition, the expression of the *LPL* gene is positively correlated with intramuscular fat content in pig muscle tissues [31]. Studies have shown that increasing IMF content can improve meat quality, including color, tenderness, flavor, and juiciness [32,33,34]. Amino acids and fatty acids are important precursors of muscle flavor, of which composition and content not only directly affect the taste and flavor of meat but also have a close relationship with human health [35,36]. Amino acid composition in meat is an important indicator for evaluating the nutritional value and quality of meat [37]. In this study, we found that overexpression of the *LPL* gene upregulated the content of all-dis-4,7,10,13,16.19- Deoosahebonoie Acid but downregulated the content of L-Glutamine and 3-Methyl-L-histidine. In addition, L-Glutamine participated in Vitamin B6 metabolism and D-Amino acid metabolism, Arginine biosynthesis, alanine, and aspartate and glutamate metabolism signaling pathways. Vitamin B6 alters blood lipid abnormalities in mice through the SIRT1/SREBP-1c pathway [38]. Arginine [39] and aspartate [40] can regulate fat deposition in animals. These results indicate that the *LPL* gene can regulate fat deposition in rabbits through L-Glutamine/multiple pathways. The results of the correlation analysis also indicate a significant negative correlation between the expression of the *LPL* gene and 3-Methyl-L-histidine content. The study also found that LPL affects the activity of histidine [41]. 3-Methyl-L-histidine is significantly negatively correlated with IMF. Histidine can promote the absorption and utilization of zinc in animals [42]. Zinc affects lipid metabolism and can reduce liver lipid deposition through autophagy [43,44]. These results indicate that the *LPL* gene may regulate IMF in rabbits through L-Glutamine, 3-Methyl-L-histidine and multiple pathways.

## 5. Conclusions

In summary, we found that METTL3 suppresses *LPL* expression in skeletal muscle and reduces circulating LPL levels during rabbit growth. During adipocyte differentiation, *METTL3* modulates *LPL* gene expression via YTHDF2-mediated recognition, thereby inhibiting the differentiation of preadipocytes. In vivo experiments further demonstrated that *METTL3* regulates *LPL* expression through *YTHDF2* in muscle tissues. Concurrently, *LPL* potentially mediated intramuscular fat by L-Glutamine, 3-Methyl-L-histidine and multiple pathways. This study lays the molecular theoretical foundation for cultivating high-quality meat rabbits.

## Figures and Tables

**Table 1:** Primers in this study.

Gene Name	Primer Sequence (5′-3′)	(Tm/°C)	(Product Size/bp)
*METTL3*	CCCACCTCAGTGGATCTGTT	60	189
	ACCCAGAGGAAGAGAAAGCC		
*LPL*	GACATTGGGGAGTTGCTGAT	60	214
	ACTTGTCGTGGCATTTCACA		
*YTHDF2*	CAGACACAGCCATTGCCTCCAC	60	122
	CCGTTATGACCGAACCCACTGC		
*β-actin*	GGAGATCGTGCGGGACAT	61.4	318
	GTTGAAGGTGGTCTCGTGGAT		
*PPARγ*	GAGGACATCCAGGACAACC	61	168
	GTCCGTCTCCGTCTTCTTT		
*FABP4*	GGCCAGGAATTTGATGAAGTC	61.4	140
	AGTTTATCGCCCTCCCGTT		
*C/EBPα*	GCGGGAACGAACAACAT	64	172
	GGCGGTCATTGTCACTGGTC		
si-*METTL3*	UCAAGGAACAACAGAGCAATT		
	UUGCUCUGUUGUUCCUUAGTT		
si-*YTHDF2*	CAUGAAUACUAUAGACCAATT		
	UUGGUCUAUAGUAUUCAUGTT		
si-*LPL*	CCUCGACAUCGAAACUAAATT		
	UUUAGUUUCGAUGUCGAGGTT		
Negative Control	UUCUCCGAACGUGUCACGUTT		
	ACGUGACACGUUCGGAGAATT		

## Data Availability

The data sets that support the findings of this study are available from the corresponding author.
